# Preoperative splenic artery embolism followed by splenectomy is safe and effective in patients with sinistral portal hypertension

**DOI:** 10.1007/s00423-021-02329-z

**Published:** 2021-09-19

**Authors:** Zihe Wang, Mao Li, Xing Huang, Junjie Xiong, Bole Tian

**Affiliations:** Pancreatology Department, West China Hospital, Sichuan University, Chengdu, China

**Keywords:** Sinistral portal hypertension, Splenic artery embolism, Splenectomy

## Abstract

**Background:**

Although preoperative splenic artery embolism (SAE) has been widely used for splenomegaly, the efficiency and safety of preoperative SAE in patients with sinistral portal hypertension (SPH) is unknown.

**Methods:**

We designed a retrospective cohort of SPH patients who received preoperative SAE in our hospital (February 2018 to September 2020) and compared to those who received splenectomy only, in terms of intraoperative and postoperative outcomes.

**Results:**

In all, 59 patients (18 patients received preoperative SAE) were analyzed. The median age was 44.7 years. Preoperative SAE reduced the intraoperative blood loss (637.0 vs. 420.3 ml, *P* = 0.041) and operation time (174.0 vs. 141.5 min, *P* = 0.012). The incidence of complications including postoperative pancreatic fistula (POPF), bleeding, and thromboembolism was comparable. Multivariate analysis showed that SAE was a protective factor for intraoperative blood loss and operation time, while prior pancreatic pseudocyst/abscess was a risk factor.

**Conclusions:**

Preoperative SAE could reduce intraoperative blood loss and operation time in SPH patients without increasing the incidence of complications compared to splenectomy only.

## Introduction

Sinistral portal hypertension (SPH), also named left-sided portal hypertension, is a rare clinical syndrome. Unlike common causes of portal hypertension, occlusion of the splenic vein results in hypertension in the drainage system for splenic blood, which further leads to the hypertension of gastric varices or esophageal varices [1]. The pathogenesis of occlusion can vary, and pancreatic disorders play a critical role [2–4].

Although most patients are asymptomatic, upper gastrointestinal bleeding has been reported in 4–17% of cases [5], and these patients might develop hemorrhagic shock and even die [2]. Thus, many treatment options including splenectomy, splenic artery embolism (SAE), and splenic vein stenting (SVS) have been proposed for fatal bleeding events [6–9]. However, in light of the common background shared by these patients—namely, a history of pancreatitis—patients with SPH usually present with severe fibrosis adjoining the spleen, which could increase the surgical difficulty and the risk of excessive blood loss. Thanks to the development of interventional therapy, a preoperative SAE method that could reduce blood flow to the spleen and consequently lower the possibility of intraoperative blood loss has been proposed [10]. Nevertheless, no studies have compared the effectiveness and safety of this new method with conventional splenectomy in SPH patients to date; thus, we were motivated to conduct this study.

We designed a retrospective cohort study to compare splenectomy only with SAE combined with splenectomy in patients with SPH to determine whether preoperative SAE could reduce intraoperative blood loss and the operation time. Secondary outcomes such as postoperative complications, expenses, and length of stay (LOS) were also analyzed.

## Material and methods

### Patients

The study had a retrospective cohort design, and patients were grouped into two cohorts on the basis of the procedures they received: splenectomy with preoperative SAE and splenectomy only. Inpatients diagnosed with SPH in the Department of Pancreatology, West China Hospital, Sichuan University (February 2018 to September 2020), were identified, and the follow-up period ended in April 2021. In all, 59 patients were selected based on the following criteria: (1) age 18 to 75 years old; (2) diagnosis of SPH based on isolated gastric or gastric and esophageal varices confirmed by endoscopy, and results of CT/MRI/DSA which inferred the primary diseases; (3) a history of gastrointestinal bleeding and failure of conservative therapies or endoscopic therapies; (4) open splenectomy performed in the Department of Pancreatology. Patients with malignant pancreatic or splenic diseases and portal hypertension derived from liver diseases were excluded. Written informed consent was obtained from all patients. The study was performed according to the Declaration of Helsinki and approved by the local ethics committee (approval number 2021–675).

### Interventional and surgical procedures

SAE procedures were performed as previously described [11]. A 2.5- or 5-Fr microcatheter via femoral artery access was advanced into the splenic artery, and the splenic artery was embolized at the level of the splenic hilum distal to the left gastroepiploic artery. Within 24 h, three experienced surgeons in our department performed open splenectomies, and they also performed the surgical procedures in the other group. Because most of the patients in both groups had a history of pancreatitis, those patients also underwent distal pancreatectomy.

### Intraoperative and postoperative outcomes

This study focused on the effect of preoperative SAE on decreasing the amount of blood loss during surgery. Considering the difficulty in precise measurement of intraoperative blood loss, we chose the equation proposed by Mercuriali et al. [12], and the blood loss in this equation was red blood cell loss per se. Therefore, intraoperative blood transfusion in this study reflected packed red cells. Complications including postoperative pancreatic fistula (POPF), bleeding (including bleeding from gastric varices and surgical sites), thromboembolism, postoperative abdominal fluid collection, pleural effusion, and adhesive ileus were all recorded during follow-up. POPF was defined by the International Study Group on Pancreatic Fistula (ISGPF) criteria [13]. Cost data represented the use of all expense categories including medications, supplies, cost of surgery, ICU staff, and cost of interventional therapy if applied.

### Statistical analysis

Continuous data that followed the normal distribution are presented as means with standard deviations (SDs), while nonnormally distributed data are presented as medians with interquartile ranges (IQRs). Categorical data are presented as frequencies and percentages. Pearson’s *χ*^2^ test or Fisher’s exact test were chosen to analyze categorical data. Two-tailed unpaired *t*-tests were employed to compare normally distributed data, whereas Mann–Whitney’s *U* tests were used for nonnormally distributed data or ranked data. Owing to the nonnormal distribution of the estimated blood loss data and operation time data, we transformed the continuous variables into binary variables based on cutoff values, defined as median blood loss and operation time in the splenectomy cohort, to perform the Firth logistic regression analysis [14]. A univariate analysis was performed to identify the potential risk factors (*P* < 0.1 for entry), and then a multivariate analysis was performed. A *P* < 0.05 denoted statistical significance. All statistical analyses were performed using RStudio 1.4 (Boston, MA).

## Results

### Demographic and clinical characteristics

A total of 59 patients were analyzed (Table 1). The median age was 44.7 years while males accounted for 72.9% (43/59) of the cohort. More patients in the preoperative SAE group (9/18) had a higher ASA score than patients in the splenectomy group (7/41) (*P* = 0.009). One hundred percent (41/41) of patients in the splenectomy group and 94.4% (17/18) of patients in the preoperative SAE group had a history of pancreatitis (*P* = 0.305). Regarding pancreatitis attacks, no significant difference was noted (*P* = 0.258), and the median number of attacks was 2 in both groups. A total of 34.1% (14/41) of patients in the splenectomy group had previously undergone abdominal surgery, while the number is 44.4% (8/18) in SAE + splenectomy group (*P* = 0.451).

**Table 1:** Demographic and clinical characteristics of the SPH patients undergoing splenectomy or SAE + splenectomy

	Total (*N* = 59)	Splenectomy (*N* = 41)	SAE + splenectomy (*N*= 18)	*P* value
Age (years), mean (SD)	44.7 (12.4)	45.0 (13.4)	44.2 (10.2)	0.825
Sex (female/male)	16/43	12/29	4/14	0.753
BMI (kg/m^2^), mean (SD)	21.3 (3.2)	21.1 (3.1)	21.7 (3.4)	0.524
ASA score, *n* (%)				
2	43 (72.8)	34 (82.9)	9 (50.0)	
3	16 (27.1)	7 (17.1)	9 (50.0)	0.009*
Prior abdominal surgery, *n* (%)	22 (37.3)	14 (34.1)	8 (44.4)	0.451
Pathological backgrounds, *n* (%)				
Pancreatic pseudocyst	33 (55.9)	22 (53.7)	11 (61.1)	0.595
Pancreatic abscess	9 (15.3)	7 (17.1)	2 (11.1)	0.708
Acute pancreatitis	2 (3.4)	1 (2.4)	1 (5.6)	0.521
Chronic pancreatitis	14 (23.7)	11 (26.8)	3 (16.7)	0.516
Gastric ulcer	1 (1.7)	0	1 (5.6)	0.305
Prior pancreatitis attack (times), median (IQR)	2 (1–4)	2 (1–4)	2 (1–2)	0.258
Albumin (g/l), mean (SD)	38.4 (6.3)	39.1 (5.8)	36.8 (7.2)	0.214
PT (s), mean (SD)	12.4 (1.2)	12.4 (1.1)	12.4 (1.5)	0.902
APTT (s), mean (SD)	29.3 (3.5)	29.8 (3.1)	28.2 (4.1)	0.097
PLT count (10^9/l), median (IQR)	142 (110–187)	152 (115–198)	136.5 (91.25–169.75)	0.344
Spleen diameter (cm), median (SD)	12.8 (12.2–13.4)	12.7 (12.1–13.4)	12.9 (11.5–14.2)	0.294
Comorbidity				
Diabetes mellitus, *n* (%)	16 (27.1)	9 (22.0)	7 (38.8)	0.212
Hypertension, *n* (%)	6 (10.2)	5 (12.2)	1 (5.6)	0.656
Hyperlipemia, *n* (%)	10 (16.9)	6 (14.6)	4 (22.2)	0.475

*SPH*, sinistral portal hypertension; *SAE*, splenic artery embolism; *SD*, standard deviation; *IQR*, interquartile range; *PT*, prothrombin time; *APTT*, activated partial thromboplastin time; *PLT count*, platelet count

*The cutoff *P* value was 0.05﻿

### Intraoperative and postoperative outcomes

Compared to patients who received preoperative SAE, patients in the splenectomy-only group significantly exhibited more blood loss (637.0 (416.5–1109.9) ml vs. 420.3 (278.1–620.1) ml, *P* = 0.041) and a longer operation time (174 (145–212) min vs. 141.5 (120–166.25) min, *P* = 0.012) (Table 2). However, a difference in intraoperative blood transfusion was not noted (splenectomy only 0 (0–600) vs. preoperative SAE 500 (0–600), *P* = 0.171). Notably, fewer patients in the SAE group (13/18) received distal pancreatectomies than patients in the splenectomy group (39/41) (*P* = 0.023). Regarding the postoperative outcomes, no deaths were reported during follow-up. The median follow-up was 18 months in the splenectomy group and 26 months in the preoperative group. POPF was the most common complication, with 22% (13/59) of patients experiencing it, yet the majority (12/13) had biochemical POPF, with only one patient in the SAE group suffering from grade B POPF (*P* = 0.146). Postoperative bleeding occurred in 5 of 59 patients. Only one patient in the preoperative SAE group reported melena during follow-up. For patients who received splenectomy, two patients presented with melena; one was diagnosed with bleeding from surgical sites, while the other one exhibited acute bleeding from postoperative stress ulcers and developed hemorrhagic shock. Thromboembolism in the portal vein developed in three patients, two patients received splenectomy only and 1 patient received preoperative SAE (4.9% vs. 5.6%, *P* = 1.000). Although one cohort received preoperative SAE, neither total LOS nor postoperative LOS showed any significant difference. In light of this finding, the cost of interventional therapy might account for the higher total cost in the SAE + splenectomy group (85,721.2 (70,800.5–104,598.3) vs. 69,358.9 (57,795.6–76,642.4), *P* = 0.002), while the surgical costs were comparable.

**Table 2:** Intraoperative and postoperative outcomes of SPH patients undergoing splenectomy or SAE + splenectomy

	Total (*N* = 59)	Splenectomy (*N* = 41)	SAE + splenectomy (*N* = 18)	*P* value
Estimated blood loss (ml), median (IQR)	502.1 (392.1–937.5)	637.0 (416.5–1109.9)	420.3 (278.1–620.1)	0.041*
Intraoperative transfusion (ml), median (IQR)	0 (0–600)	0 (0–600)	500 (0–600)	0.171
Operation time (min), mean (IQR)	160 (130–200)	174 (145–212)	141.5 (120–166.25)	0.012*
Distal pancreatectomy, *n* (%)	52 (88.1)	39 (95.1)	13 (72.2)	0.023*
Pseudocyst resection, *n* (%)	2 (3.4)	1 (2.4)	1 (5.6)	0.521
Pseudocyst drainage, *n* (%)	31 (52.5)	21 (51.2)	10 (52.6)	0.759
Abscess drainage, *n* (%)	9 (15.3)	7 (17.1)	2 (10.5)	0.708
Complications, *n* (%)				
No POPF	46 (78.0)	34 (82.9)	12 (66.7)	
Biochemical POPF	12 (20.3)	7 (17.1)	5 (27.8)	
POPF B	1 (1.7)	0	1 (5.6)	0.146
Bleeding	5 (8.5)	4 (9.8)	1 (5.6)	1.000
Thromboembolism	3 (5.1)	2 (4.9)	1 (5.6)	1.000
Pleural effusion	2 (3.4)	1 (2.4)	1 (5.6)	0.521
Infected abdominal fluid collection	7 (11.9)	4 (9.8)	3 (16.7)	0.664
Noninfected abdominal fluid collection	2 (3.4)	2 (4.9)	0	1.000
Adhesive ileus	2 (3.4)	1 (2.4)	1 (2.4)	0.521
Total LOS (days), median (IQR)	13 (9–18)	13 (10–17)	12.5 (9–19)	0.791
Postoperative LOS (days), median (IQR)	6 (6–8)	6 (6–8)	8(5–10.3)	0.159
Total cost^a^ (RMB), median (IQR)	71,167.4 (58,779.3–86,028.7)	69,358.9 (57,795.6–76,642.4)	85,721.2 (70,800.5–104,598.3)	0.002*
Surgery cost (RMB), mean (SD)	23,381.7 (6278.5)	23,604.6 (5784.4)	22,874.0 (7441.3)	0.684
Interventional therapy cost (RMB), mean (IQR)	NS	NS	17,774.5 (13,014.7–21,368.0)	NS

*SPH*, sinistral portal hypertension; *SAE*, splenic artery embolism; *SD*, standard deviation; *IQR*, interquartile range; *POPF*, postoperative pancreatic fistula; *LOS*, length of stay; *NS*, not suitable; *RMB*, the currency Chinese Yuan

^a^Total cost included medications, supplies, nursing service, surgery cost, ICU cost, and cost of interventional therapy if applied

*The cutoff *P* value was 0.05

### Risk factors for excessive intraoperative blood loss in patients with sinistral portal hypertension who underwent splenectomy

We conducted univariate and multivariate analyses using demographic and clinical characteristics and intraoperative variables to identify risk factors for intraoperative blood loss (Table 3). In the univariate analysis, factors associated with excessive intraoperative blood loss included prior abdominal surgery, prior pancreatic pseudocyst/abscess, preoperative albumin, PT, APTT, intraoperative transfusion, and preoperative SAE. In the multivariate analysis, only preoperative SAE (OR 0.136, 95%CI 0.019–0.652) was identified as a protective factor, while prior pseudocyst/abscess was a risk factor (OR 6.734, 95%CI 1.411–48.923).

**Table 3:** Univariate and multivariate analysis of the predictors of excessive blood loss in SPH patients who underwent splenectomy

	Univariate analysis		Multivariate analysis	
	Odds ratio (95% CI)	*P*	Odds ratio (95% CI)	*P*
Age	0.991 (0.950–1.034)	0.666		
Sex (female)	1.742 (0.516–5.878)	0.371		
BMI	1.119 (0.947–1.323)	0.187		
ASA score	1.190 (0.372–3.801)	0.770		
Prior abdominal surgery	2.500 (0.844–7.401)	0.098*	1.331 (0.302–5.655)	0.697
Prior pancreatitis attack	0.978 (0.883–1.084)	0.676		
Prior pancreatic pseudocyst/abscess	8.250 (1.674–40.652)	0.010*	6.734 (1.411–48.923)	0.015*
Albumin	0.905 (0.824–0.993)	0.035*	1.016 (0.877–1.190)	0.828
PT	1.730 (1.065–2.811)	0.027*	1.294 (0.656–2.807)	0.459
APTT	1.173 (0.992–1.387)	0.061*	1.017 (0.821–1.269)	0.876
PLT count	1.002 (0.996–1.008)	0.468		
Spleen diameter	0.945 (0.745–1.198)	0.639		
Operation time	1.001(0.991–1.010)	0.886		
Distal pancreatectomy	4.759 (0.534–42.375)	0.162		
Intraoperative transfusion	1.003 (1.001–1.005)	0.002	1.003 (1.000–1.007)	0.017*
Preoperative SAE	0.300 (0.084–1.067)	0.063*	0.088 (0.008–0.495)	0.004*

*SPH*, sinistral portal hypertension; *PT*, prothrombin time; *APTT*, activated partial thromboplastin time; *PLT count*, platelet count; SAE, splenic artery embolism

Excessive blood loss defined as > 637.0 ml (median estimated blood loss of splenectomy group)

The cutoff *P* value was 0.1 in the univariate analysis and 0.05 in the multivariate analysis

*The cutoff *P* value was 0.05

### Risk factors for prolonged operation time in patients with sinistral portal hypertension who underwent splenectomy

We also analyzed the risk factors for prolonged operation time (Table 4). Prior abdominal surgery and prior pancreatic abscess/pseudocyst were identified as risk factors in both the univariate and multivariate analyses (OR 3.615, 95%CI 1.147–12.709; OR 4.559, 95%CI 1.215–21.468), while preoperative SAE was a protective factor (OR 0.185, 95%CI 0.039–0.676).

**Table 4:** Univariate and multivariate analysis of the predictors of prolonged operation time in SPH patients who underwent splenectomy

	Univariate analysis		Multivariate analysis	
	Odds ratio (95% CI)	*P*	Odds ratio (95% CI)	*P*
Age	1.024 (0.980–1.070)	0.286		
Sex (female)	0.762 (0.238–2.443)	0.647		
BMI	1.001 (0.850–1.180)	0.987		
ASA score	1.867 (0.583–5.975)	0.293		
Prior abdominal surgery	2.836 (0.948–8.487)	0.062*	3.370 (1.021–12.409)	0.046*
Prior pancreatitis attack	1.083 (0.967–1.212)	0.170		
Prior pancreatic pseudocyst/abscess	4.242 (1.061–16.968)	0.041*	4.559 (1.215–21.468)	0.024*
Spleen diameter	0.905 (0.710–1.153)	0.418		
Distal pancreatectomy	1.694 (0.300–9.561)	0.551		
Intraoperative transfusion	1.001 (1.000–1.002)	0.192		
Preoperative SAE	0.210 (0.053–0.837)	0.027*	0.154 (0.031–0.586)	0.005*

*SPH*, sinistral portal hypertension; *SAE*, splenic artery embolism

Prolonged operation time defined as > 174 min (median operation time of splenectomy group)

The cutoff *P* value was 0.1 in the univariate analysis and 0.05 in the multivariate analysis

*The cutoff *P* value was 0.05

## Discussion

Portal hypertension is a well-discussed topic, and its causes can be groups as prehepatic, hepatic, and posthepatic while hepatic (mainly cirrhosis) is the most common one. However, for patients with SPH, the etiology evidently differed from those with hepatic portal hypertension. Considering that clinical manifestations of SPH mainly come from primary diseases and splenic vein occlusion, splenectomy is preferred for patients who present with gastrointestinal bleeding. Based on our experience, a history of pancreatitis could give rise to a greater risk of adhesions in the abdominal area, making splenectomy for SPH patients much more difficult than that for patients with other causes of splenomegaly. Thus, a safer, more effective procedure must be developed.

With surgeons at the crossroads of treatment, interventional therapy has come to the center stage. Wang and colleagues conducted a study of 14 patients with SPH, and reported no severe complications, such as splenic abscess or rupture of the spleen [15]. However, they also admitted that postembolization syndrome was the most frequent complication, although they did not report the exact number of events. Splenic vein stenting was also proposed to be an effective and safe operation for SPH patients with gastrointestinal bleeding, and it was proven to be more effective than SAE in the control of rebleeding [16]. Notably, 17.4% (4/23) and 7.1% (1/14) of patients in the SAE group and the SVS group, respectively, required subsequent splenectomy due to rebleeding. The study by Fernandes et al. also reported that a high proportion (3/5) of patients who received SAE were submitted to splenectomy finally, even though the sample size was small [3]. In fact, splenic artery embolism was also reported to cause various kinds of complications—not only postembolization syndrome, but also splenic abscess, splenic vein thrombosis, and pancreatitis. Splenectomy, as a surgical approach, could efficiently reduce the risk of further hemorrhage by decreasing venous outflow through the collateral circulation and associated varices. Notably, surgeons may also need to address primary pancreatic diseases when performing splenectomy. In light of that, splenectomy might still be preferable due to its effectiveness; meanwhile, for those patients in poor conditions and cannot bear surgery, the choice of interventional therapies might be preferred.

Under these circumstances, the combination approach of surgical and interventional therapy finally drew the attention of surgeons. Preoperative SAE was first proposed by Poulin et al. in 1993 [10]. This method was meant to control arterial flow and lower the risk of intraoperative bleeding, although it might also cause thrombosis in the venous vessels. In addition, it has been reported to decrease the splenic volume, thereby improving the surgical view and intraoperative exposure [17]. Reso and colleagues performed 19 laparoscopic splenectomies after SAE in patients with massive splenomegaly and reported a median estimated blood loss of 200 ml and a median operation time of 130 min [18]. In addition, the results of one comparative study implied a shorter operation time, less estimated blood loss, and shorter postoperative hospital stay for patients who received preoperative SAE [11]. It might raise the question of why the intraoperative blood loss in those studies was less than ours (637.0 ml for the splenectomy-only group and 420.3 ml for the preoperative SAE group). We believed that differences in the primary disease and choice of open surgery could account for this discrepancy. As we mentioned, 98.3% (58/59) of patients in our study suffered from pancreatitis, which resulted in severe fibrosis in the abdominal cavity and might lead to more intraoperative blood loss and a prolonged operation time. In addition, we performed open splenectomy on all patients in this study since portal hypertension was a contraindication for laparoscopic splenectomy. It was widely believed that open splenectomy could cause more intraoperative blood loss than laparoscopic splenectomy.

There was one notably significant difference observed between the patients: more patients in the splenectomy-only group received distal pancreatectomy (39/41 vs. 13/18, *P* = 0.023), which might raise questions regarding whether this discrepancy could lead to a prolonged operation time and more blood loss. In our hospital, the diseased pancreas is divided with a stapler so that the tail of the pancreas and the spleen can be resected at almost the same time. In other words, distal pancreatectomy did not take a longer time, which was supported by the results of logistic regression analysis. Furthermore, we also conducted an analysis with patients who received distal pancreatectomy, and proved the effectiveness of preoperative SAE in reducing operation time (174 (145–212) vs. 148 (125–167.5) min, *P* = 0.046)) and blood loss (662.2 (421.4–1120.7) ml vs. 428.5 (326.8–600) ml, *P* = 0.048).

The incidence of some complications reported in this study notably differed from others. Compared to studies that performed preoperative SAE and splenectomy, this research reported that more patients developed postoperative abdominal fluid collection (9/59), but fewer developed pleural effusion (2/59) [18]. We thought differences in the pathological backgrounds might still be a crucial reason for these differences among studies. Some of our patients underwent drainage of pancreatic pseudocysts due to a history of pancreatitis, and the fibrosis in abdominal cavity might increase the risk of fluid collection postoperatively. In our study, the incidence of postoperative bleeding was 9.8% (4/41) and 5.6% (1/18) in the splenectomy-only group and preoperative SAE group, respectively. Two patients presented with bleeding due to stress ulcers and bleeding from the surgical site, which should not be interpreted as rebleeding from gastric varices. The occurrence of rebleeding among patients with SPH in other studies varied from 0 to 17.4% [3, 6, 15, 16, 19–21], while the choice of therapy included observation, splenectomy, SAE, and SVS. It should be clarified that many studies had a relatively small sample size and included patients without gastrointestinal bleeding, which increased the difficulty in comparing the effectiveness.

There were some limitations to this study. First, it was retrospectively designed, and thus, selection bias may exist. However, there was no notable difference in the demographic and clinical characteristics, except for the ASA score, with more patients in preoperative SAE group seeming to have higher scores. Since preoperative SAE could not remain concealed, it was also challenging to implement blinding. Second, the size of the sample is small de facto. Only 18 patients received preoperative SAE, which might have caused relatively high heterogeneity, but we also have to point out that, to our knowledge, this study is the first to compare preoperative SAE to splenectomy only in SPH patients. Finally, we excluded patients with pancreatic or splenic malignancy because these diseases would require other surgical methods that might interfere with the analysis of outcomes such as intraoperative blood loss and operation time. Further studies with a larger sample size and stratified analysis might provide more supportive evidence.

## Conclusions

This study demonstrated that preoperative SAE could efficiently reduce intraoperative blood loss and the operation time for patients with sinistral portal hypertension when undergoing splenectomy. Preoperative SAE did not increase the incidence of complications (POPF, bleeding, abdominal fluid collection, pleural effusion, and thromboembolism) or mortality among these patients. Since the LOS and surgery costs were comparable between the two groups, patients who received preoperative SAE had higher overall healthcare expenses.

## Data Availability

The authors confirm that the data supporting the findings of this study are available within the article.

## Code availability

RStudio 1.4 was used.
